# *Sulfolobus* Mutants, Generated via PCR Products, Which Lack Putative Enzymes of UV Photoproduct Repair

**DOI:** 10.1155/2011/864015

**Published:** 2011-07-07

**Authors:** Cynthia J. Sakofsky, Laura A. Runck, Dennis W. Grogan

**Affiliations:** Department of Biological Sciences, University of Cincinnati, 614 Rieveschl Hall, Clifton Court, Cincinnati, OH 45221-0006, USA

## Abstract

In order to determine the biological relevance of two *S. acidocaldarius* proteins to the repair of UV photoproducts, the corresponding genes (Saci_1227 and Saci_1096) were disrupted, and the phenotypes of the resulting mutants were examined by various genetic assays. The disruption used integration by homologous recombination of a functional but heterologous *pyrE* gene, promoted by short sequences attached to both ends *via* PCR. The phenotypic analyses of the disruptants confirmed that ORF Saci_1227 encodes a DNA photolyase which functions *in vivo*, but they could not implicate ORF Saci_1096 in repair of UV- or other externally induced DNA damage despite its similarity to genes encoding UV damage endonucleases. The success of the gene-disruption strategy, which used 5′ extensions of PCR primers to target cassette integration, suggests potential advantages for routine construction of *Sulfolobus* strains.

## 1. Introduction

Gene inventories of hyperthermophilic archaea (HA) suggest that this deeply branching clade of prokaryotes may employ unusual molecular strategies during DNA replication and repair. For example, HA have certain DNA enzymes and enzymatic properties not found in mesophilic archaea or bacteria. These include reverse DNA gyrase, a type I topoisomerase that introduces positive superhelical turns into DNA [1], and family-B DNA polymerases which stall specifically ahead of dU residues in the template strand [2, 3]. Conversely, HA lack certain DNA-repair proteins that are widely conserved in other organisms; they encode no homologues of DNA mismatch repair proteins MutS and MutL [4], and they also lack the UvrABC homologues that mediate nucleotide excision repair (NER) in bacteria and mesophilic archaea [5]. Whereas HA do encode homologues of the eukaryotic helicases and structure-specific nucleases that complete the process of NER in eukaryotes, they do not have the corresponding proteins that initiate NER. The fact that no HA encode homologues of known NER-specific damage-recognition proteins seems significant, since these proteins are required for NER function in other organisms, and are the only proteins specific to NER in eukaryotes. 

Experimental data, nevertheless, suggest that HA can excise UV photoproducts from DNA although the mechanism and proteins responsible have not been identified. Biochemical assays indicate time-dependent loss of cyclobutane pyrimidine dimers from genomic DNA of intact *Sulfolobus solfataricus* cells at physiological temperature [6, 7]. In* Sulfolobus acidocaldarius* conjugation assays, UV enhances recombinant formation, suggesting conversion of pyrimidine dimers, which are not recombinogenic *per se,* into lesions that increase intercell transfer of DNA, recombination, or both. Also, the effect decays at physiological temperatures with kinetics similar to those of the concentration of UV photoproducts in *S. solfataricus* [6–8].

In addition, two *S. acidocaldarius* genes encode proteins predicted to repair UV photoproducts independently of each other and of NER. The first is a putative DNA photolyase encoded by ORF Saci_1227. The *Sulfolobus tokodaii* homologue of this protein photoreactivates DNA *in vitro* [9]. *S. acidocaldarius* exhibits efficient photoreactivation *in vivo* [10], but the gene product responsible for this has not been established experimentally. The second protein, encoded by Saci_1096, belongs to a family of known and putative UV-damage endonucleases (UVDEs). The best-characterized of these enzymes occur in the fission yeast *Schizosaccharomyces pombe* and the radioresistant bacterium *Deinococcus radiodurans*, where they mediate an alternative excision repair pathway for UV photoproducts [11, 12]. In this alternative pathway, the phosphodiester backbone is cut on the 5′ side of the UV photoproduct, and the lesion is removed by strand-displacement synthesis [11].

As others have suggested, hyperthermophilic archaea could, in principle, recruit an enzyme with affinity for UV photoproducts to serve as a photoproduct-recognition protein for an NER-like repair of these lesions (although this would not address other forms of DNA damage) [6]. DNA photolyase seems a logical candidate for this role, as it should bind to UV photoproducts in the dark but remain unable to repair them under these conditions. Also, cooperation between these distinct repair systems has precedent in that photolyase enhances repair of UV photoproducts by the NER proteins of *E. coli* [13].

As the most direct route to clarifying the biological roles of these two *S. acidocaldarius* proteins, we were interested in disrupting the corresponding genes and characterizing the mutants phenotypically. Several genes in *S. solfataricus* and related *Sulfolobus* species have been disrupted by inserting a functional *pyrE* gene between segments of *Sulfolobus* chromosome (typically several hundred base pairs long) cloned in a plasmid vector. The resulting plasmid is then transformed into a *pyrE* recipient, and the selected gene integrates into the *Sulfolobus* chromosome through homologous recombination [14–16]. Although we have found this approach to be effective for genes of *Sulfolobus acidocaldarius*, published studies show that the recombination system of *S. acidocaldarius *can also operate on much shorter DNA sequences, that is, down to 30 bp or less [17–19]. This suggested that the need for a plasmid construct may be avoided if PCR primers were used to attach short flanking (i.e., targeting) sequences to the ends of a selected marker, a technique that has been used extensively in *Saccharomyces cerevisiae* [20].

In the present study, we demonstrated the feasibility of the PCR-tailing approach for *S. acidocaldarius* genetics by disrupting both the Saci_1227 and Saci_1096 genes individually by this method. We went on to characterize the mutants by measuring phenotypic properties related to repair of UV photoproducts and other DNA damage. The results indicate that Saci_1227 encodes a functional DNA photolyase of *S. acidocaldarius *and that neither this protein nor that encoded by Saci_1096 are essential for dark repair of UV photoproducts.

## 2. Methods and Materials

### 2.1. Strains, Cultivation, and Genetic Manipulations

The *S. acidocaldarius* pyrimidine auxotroph MR31 [21] was grown in xylose-tryptone medium supplemented with 20 mg uracil per liter, as described previously [22]. Cells were washed, cryo-preserved, and transformed by electroporation [23]. The linear DNA used for gene targeting was generated from pLK3a by PCR (see below). Increased length of the product (reflecting incorporation of targeting sequences) was confirmed by agarose gel electrophoresis, and unincorporated primers were removed using centrifugal membrane concentrators. Pyr^+^ transformants were selected by spreading electroporation mixtures onto plates of xylose-tryptone medium lacking uracil. Colonies appearing after one week incubation were streaked for isolation on selective (uracil-free) medium, and the genotype was confirmed by PCR analyses (see Section 3).

To restore the function of the native *S. acidocaldarius pyrE* gene in the disruptants, spontaneous *pyrE*_Sso_ mutants of these strains were first selected on plates supplemented with 5-fluoro-orotic acid (FOA, 50 mg per liter) and uracil; this takes advantage of the fact that only *Sulfolobus* mutants lacking either *pyrE* or *pyrF* function are resistant to FOA. After clonal purification, the genetic stability of the mutants was tested by spreading about 10^8^ cells on uracil-free plates. Auxotrophs confirmed to be stable were identified and electroporated with DNA of plasmid pSAPE5, which carries the intact *pyrE*_Sac_ gene [18]. The resulting Pyr^+^ transformants were confirmed to have a fully restored *pyrE*_Sac_ gene by PCR and sequencing. 

### 2.2. DNA Constructions

To construct plasmid pLK3a, which contains the *pyrE*_Sso_ gene cloned into pNEB193, *pyrE*_Sso_ was amplified from pMJ03 [24] using Phusion DNA polymerase (Finnzymes OY, Espoo, Finland) and primers SsoPEFAvrKpnf1 and SsoPEAvrKpnr1 (Table 1) which added flanking AvrII and KpnI restriction sites to both ends of the PCR products. The program used initial denaturation at 98°C for 30 s, followed by 28 cycles of 98°C for 7 s, 64°C for 20 s, 72°C for 45 s, and final extension at 72°C for 7 min. Agarose gel electrophoresis confirmed a product of the expected size (0.7 kb), corresponding to *pyrE*_Sso_ and associated promoter. This PCR product was then digested with AvrII and was ligated to pNEB193 previously digested with XbaI and Antarctic phosphatase (New England Biolabs, Beverly, Mass, USA). The resulting plasmid extracted from a transformant was confirmed to contain the *pyrE*_Sso_ gene and to lack XbaI and AvrII sites; this construct was designated pLK3a. 

To construct a repair-proficient *S. acidocaldarius* strain for the comparison to the initial disruptants, the* S. acidocaldarius trpC *gene (Saci_1427) cloned into the MCS of pUC19 (construct pPCBE12; see Table 1) was disrupted by an insertion of *pyrE*_Sso_ as follows. Plasmid pPCBE12 was digested with SpeI for 1 hr at 37°C and heat inactivated at 65°C for 20 minutes. The mixture was then treated with Antarctic Phosphatase (New England Biolabs, Beverly, MA) and heat inactivated at 65°C for 5 mins. The *pyrE*_Sso_ gene was amplified from pMJ03 with flanking AvrII restriction sites. This product was digested with AvrII for 1 hr at 37°C and ligated into the *trpC* plasmid to generate pLK4a (Table 1).

### 2.3. Assays of Sensitivity

Measurements of UV survival and photoreactivation followed the procedures of previous studies [10, 25]. Growth inhibition by chemical agents was evaluated in a series of 0.15-mL cultures containing successive 1 : 2 dilutions of the test compound. Each culture was inoculated with about 10^6^ cells, and growth was scored after three days incubation at 78°C–80°C to determine the minimum inhibitory concentration (MIC) of the test compound. Survival of ionizing radiation was assayed as described previously [17]; specifically, cells were suspended in sterile dilution buffer and exposed to a spatially uniform flux of gamma rays from a ^60^Co source (University of Cincinnati Nuclear Engineering facility).

### 2.4. Genetic Assays

The rate of spontaneous* pyrE* mutation was determined as described previously by growing 25–30 independent liquid cultures and plating each in its entirety on the uracil plus FOA medium described above. The average number of cells per culture for each set of fluctuation tests was determined by serial dilutions and plating of 3-4 cultures from the set, and the mutation spectrum was sampled by picking one mutant from each independent culture and sequencing its *pyrE* gene, as described previously [26]. To minimize quantitative variability, the phenotypically leaky frameshift mutations at bp 545–551 [26] were not included in the spectral analyses.

Conjugation and recombination was measured by the “marker exchange” assay [27] in which equal numbers of cells of two genetically distinct *pyr* mutants are mixed and spread on plates of uracil-free medium. The number of Pyr^+^ colonies that formed was normalized to the number of viable cells plated and was corrected for any reversion observed for each strain plated separately. Stimulation of marker exchange (SME) was measured by performing the UV exposure and all subsequent steps under dim red light to prevent photoreactivation [25]. Similarly, the decay of SME at physiological temperature was measured by holding the irradiated cell suspensions in the dark at 70°C for various lengths of time before mixing and plating, as described previously [8].

### 2.5. Calculations

Rates of spontaneous mutation to FOA resistance were calculated using the Ma-Sandri-Sarkar maximum likelihood estimator (MSS-MLE) [28], as implemented by the FALCOR web interface [29]. Input parameters were the FOA-resistant colony counts from each independent culture of the set (**r**) and the average number of viable cells per culture (**N**). Differences between mutation rates were evaluated statistically using one-way analysis of variance (ANOVA). The samples of mutation spectra drawn from the corresponding strains were compared for statistically significant differences using the hyper-G program of Cariello et al. [30].

## 3. Results

### 3.1. Gene Disruption

As the background in which to disrupt *S. acidocaldarius* genes, we chose *pyrE* mutant MR31 because of several genetic properties: (i) the mutation in this strain (*pyrE*131) removes 18 bp of *pyrE* and, therefore, does not revert, (ii) MR31 shows no residual growth on uracil-free media supplemented with tryptone, and (iii) the strain exhibits normal OMP decarboxylase levels. The last property contrasts with the majority of spontaneous *pyrE* mutations in *S. acidocaldarius*, because most of them are frame shifts and exert polar effects on *pyrF* expression [21]. The lack of polarity of *pyrE*131 offers the advantage that uracil auxotrophy can be complemented fully in *trans* using only a functional *pyrE* gene, which simplifies construction of the disrupting unit.

**Figure 1:**
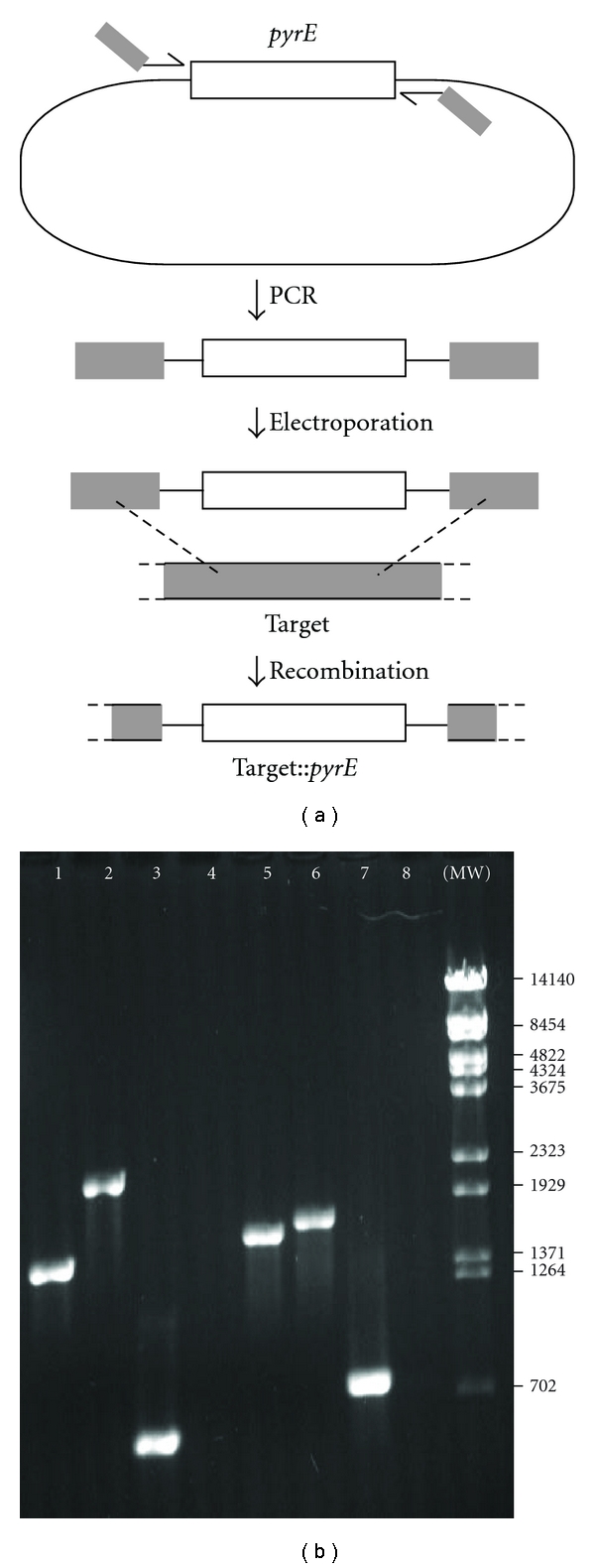
PCR analysis of disruption mutants LR10 and LR12. (a) Outline of the gene-disruption scheme. A plasmid-borne *pyrE* gene served as template for PCR, which attached *S. acidocaldarius* chromosomal sequences (gray regions at 5′ ends of the primers) to the ends of this selectable cassette. After the resulting linear DNA was introduced into *S. acidocaldarius* cells, homologous recombination (indicated by dashed lines) replaced the targeted gene with the cassette. (b) Confirmation of mutant strain genotypes. PCR reactions used genomic DNAs of wild-type *S. acidocaldarius* (lanes 1, 3, 5, and 7), Saci_1227 disruptant LR10 (lanes 2 and 4), or Saci_1096 disruptant LR12 (lanes 6 and 8) with the following primers: Saci1227 (whole-gene), lanes 1 and 2; Saci1227 internal, lanes 3 and 4; NXSaci1096 (whole-gene), lanes 5 and 6; NXSaci1096 internal, lanes 7 and 8 (see Table 1 for primer data). MW: Molecular weight marker (*λ* DNA digested with BstEII); fragment lengths (in bp) shown in the right-hand margin.

As summarized schematically in Figure 1(a), plasmid pLK3a provided a source of a functional but divergent *pyrE* gene (*pyrE*_Sso_). This gene was amplified by standard PCR using bipartite primers (SacPhr::cassf1 and SacPhr::cassr1, Saci1096 DTf3 and Saci1096 DTr3; see Table 1) in which the 3′ ends annealed to pLK3a, and the 5′ end (41–50 nt, depending on the primer) corresponded to sequences near the boundaries of ORFs Saci_1227 and Saci_1096. This resulted in PCR products predicted to replace the corresponding *S. acidocaldarius* genes by homologous recombination. Specifically, the primers were designed to replace about 620 bp of the 1293-bp putative photolyase gene (Saci_1227) with a 1498-bp insert encoding the *pyrE*_Sso_ gene and flanking sequences from pLK3a, and similarly, to replace 825 bp of the 870 bp putative UVDE gene (Saci_1096) with a segment of 1047 bp that included the *pyrE*_Sso_ gene. 

Each of these PCR products were electroporated into MR31 cells, and the cells were plated on selective (uracil-free) medium. Pyr^+^ transformants were streaked for isolation on the same medium, and DNA extracted from the pure cultures was analyzed by PCR and sequencing. The results confirmed that the Saci_1227 and Saci_1096 loci were enlarged in the transformants by the amount expected for replacement of the central region of each gene by the *S. solfataricus pyrE* gene and associated vector sequences (Figure 1(b), lane 1 versus 2, lane 5 versus 6). PCR using internal primers further confirmed that each Pyr^+^ transformant had lost all functional copies of the targeted gene (Figure 1, lane 3 versus 4, lane 7 versus 8). Additional PCR (not shown) also detected the expected orientation and spacing of juxtaposed *pyrE*_Sso_ cassette and *S. acidocaldarius* genomic sequences, and sequencing of the products revealed junctions represented in the bipartite PCR primers. The Saci_1227 and Saci_1096 disruption strains confirmed by these criteria were designated LR10 and LR12, respectively (Table 1).

### 3.2. Phenotypic Tests

**Figure 2:**
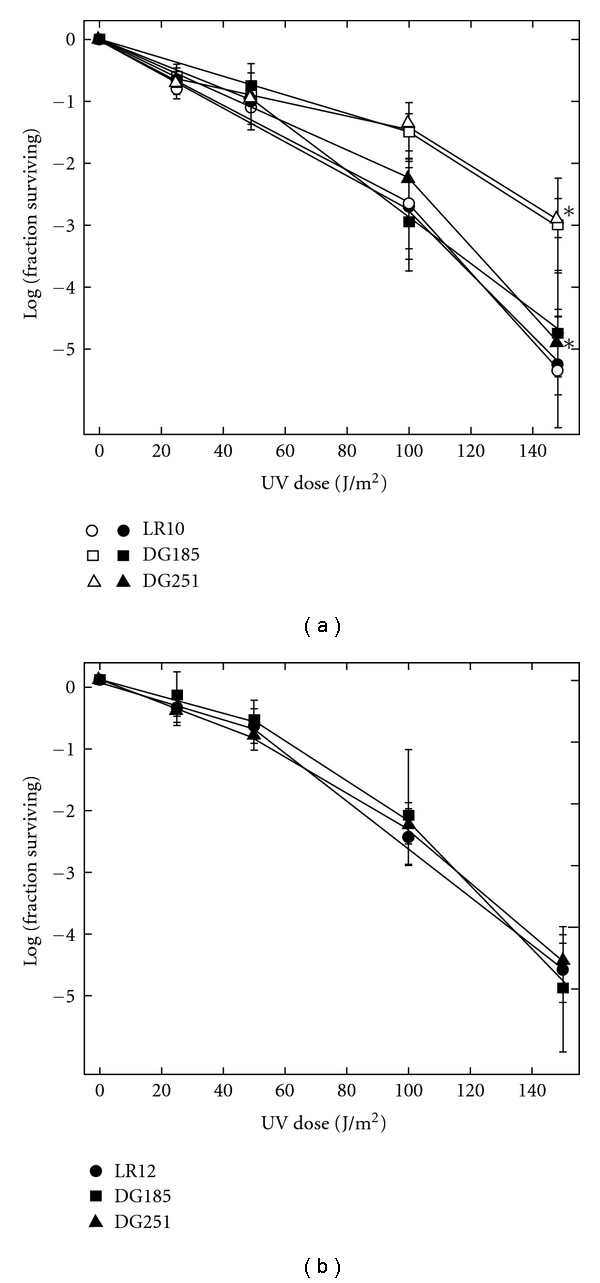
UV survival. (a) Suspensions of LR10 (circles), DG185 (squares), and DG251 cells (triangles) were irradiated with the indicated dose of UV-C and plated for viability. Open symbols indicate post-UV exposure to white light; asterisks indicate statistically significant differences. (b) LR12 (circles), DG185 (squares) and DG 251 (triangles). Exposure conditions as in (a), except that samples were not photoreactivated.

Photoreactivation of strain LR10 was evaluated in parallel with positive controls DG185 (wild type), and DG251, a *trpC*::*pyrE*_Sso_ disruptant. Each suspension of washed cells was irradiated with a defined dose of UV-C light (*λ* < 300 nm) from a germicidal lamp and was then divided into two equal portions. One portion was kept in the dark, and the other was illuminated 1 h under an array of standard fluorescent bulbs producing broad-spectrum visible light with an incident intensity of about 10 W/m^2^. As indicated by relative survival (illuminated versus dark-held), white light had no effect on untreated control cells, but increased the survival of cells previously exposed to UV-C, and the effect of the white light became more pronounced at higher UV doses (Figure 2(a)). At the highest UV dose, white-light illumination of LR10, DG185, and DG251 suspensions increased viability by a factor of 1.5, 22.2, and 91, respectively. Thus, of the three strains tested in parallel under these conditions, only the Saci_1227 disruption did not exhibit photoreactivation. This demonstration of the Phr^−^ phenotype, therefore, indicated that Saci_1227 encodes the primary functional DNA photolyase of *S. acidocaldarius*.

The impact of the Saci_1096 disruption on UV survival was also tested under these conditions, but without any white-light illumination after UV. No significant change in UV survival was detected in the disruption strain LR12 despite the wide range of UV doses leading to an overall survival of about 10^−4^ at the highest dose (Figure 2(b)). This result indicated that the Saci_1096 protein may not repair photoproducts in the *S. acidocaldarius* chromosome.

To investigate whether the Saci_1096 gene product may play a role in repairing other DNA lesions, we measured the MIC values of several chemicals for strain LR12 and the control strains in parallel assays. The compounds were chosen to produce diverse DNA lesions, ranging from intra-and interstrand DNA crosslinks, to oxidized or alkylated bases, to bulky aromatic adducts. Under these conditions, the disruption strain showed sensitivities indistinguishable from those of the control strains for all agents tested (Table 2). In other tests, suspensions of the disrupting and control strains were exposed to a high dose (150 kRad) of gamma radiation. Subsequent plating yielded average survival of 1.7 × 10^−5^ for the disruptant and 9.4 × 10^−6^ for the control strain. We were thus unable to identify a form of DNA damage that affected the Saci_1096 disruptant more than the control strains.

### 3.3. Assays of Mutagenesis and DNA Repair In Vivo

In light of these results, we examined biological functions of the Saci_1227 and Saci_1096 gene products that may not measurably alter sensitivity to DNA-damaging agents. Although *S. acidocaldarius* supports quantitative assays for mutation, DNA transfer, and homologous recombination, these assays typically involve the native *pyrE*_Sac_ gene, which in strains LR10 and LR12 had been inactivated by deletion and was complemented in *trans* by a heterologous gene (*pyrE*_Sso_). To enable the full capabilities of these assays, we, therefore, restored function of the native *pyrE*_Sac_ gene in the original disruptants by a two-stage procedure. First, spontaneous FOA-resistant mutants of LR10 and LR12 were isolated and confirmed to contain stable mutations of the heterologous *pyrE*_Sso_ gene by reversion tests and sequencing. Then, these stable Pyr^−^ mutants were transformed to uracil prototrophy with the functional *pyrE*_Sac_ gene of plasmid pSAPE5 [18]. The native *pyrE*_Sac_ gene was amplified from the chromosome and sequenced, confirming that the 18 bp deleted from this locus in MR31 had been fully restored. The corresponding LR10 and LR12 derivatives were designated CS1 and CS3, respectively (Table 1).

Construction of strains CS1 and CS3 enabled the roles of the Saci_1227 and Saci_1096 gene products in spontaneous mutation to be evaluated using the native *pyrE*_Sac_ gene [26]. Experimentally determined rates of spontaneous mutation (±standard deviation) for strains CS1, CS3, and DG185 were 4.4 (±1.9) × 10^−7^, 4.8 (±2.2) × 10^−7^, and 2.8 (±1.4) × 10^−7^ events per cell division, respectively. As confirmed by ANOVA, these fluctuation tests could not establish statistically significant differences in overall mutation rate.

**Figure 3:**
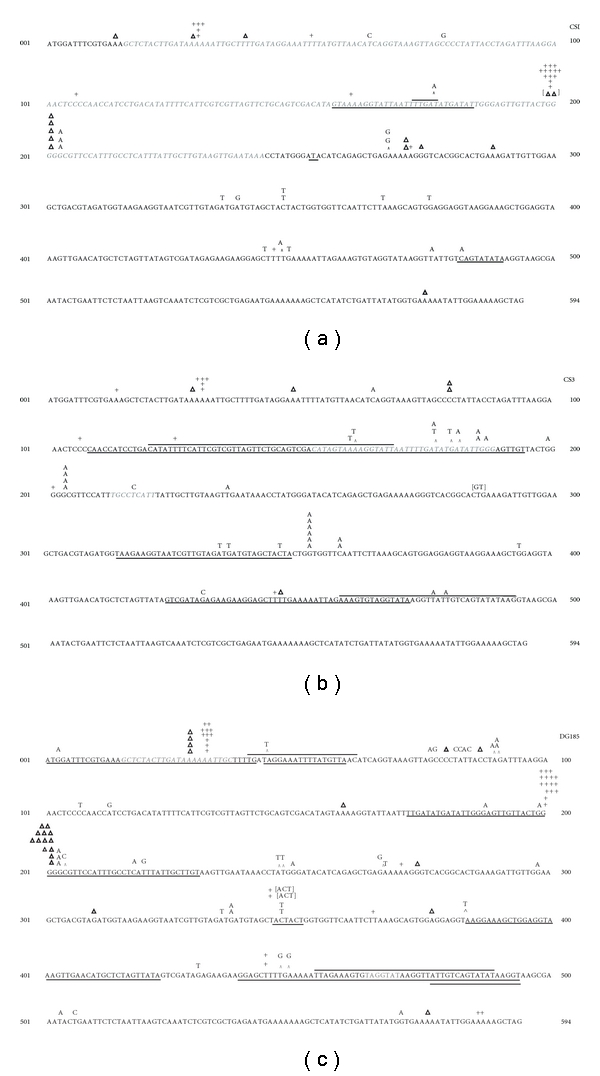
Spectra of spontaneous *pyrE* mutations in disruptant and control strains. The sequence is of the *S.acidocaldarius pyrE* coding region, and markings indicate the spontaneous mutations recovered from (a) CS1 (b) CS3 and (c) DG185. Regions removed in deletion mutations are represented by gray italicized text. Tandem duplications (>3 bp) are indicated by an underline or overline. A “+” or a “Δ” sign above a mononucleotide run denotes a single base insertion or deletion, respectively, of the base below it. Placement of these symbols above mononucleotide runs is arbitrary, as all positions of a run are genetically equivalent. Single base pair insertions not located at mononucleotide runs are shown by a “*∧*” sign with the insertion above it. Bracketed text indicates mutations occurring in a single mutant.

To analyze the molecular nature of spontaneous mutation in these strains, we sampled the corresponding mutation spectra by sequencing one randomly chosen mutant per independent culture of the fluctuation tests. The sequence changes, and positions at which they occurred, appear to be broadly similar for all three strains (i.e., wild type, Saci_1227, and Saci_1096 disrupted) (Figure 3). Comparing the major classes of mutation represented in the three spectra suggested subtle differences among the strains, however (Table 3). For example, the CS3 spectral sample exhibited about two-thirds the number of −1 frameshift mutations and 1.5 times as many base pair substitutions as wild-type and Phr^−^ strains. We, therefore, compared each of the disruptant mutation spectra to the wild-type spectrum, using the Monte Carlo estimation for Fisher's exact hypergeometric test [30]. For these tests, we evaluated several criteria for binning the mutations of Figure 3; the two criteria producing the lowest *P* values were (i) the position of the mutation within the gene and (ii) the molecular nature of the mutation. These analyses (Table 3) did not find statistical significance of spectral differences at the *P* = .05 level, but the *P* values of the CS3 spectrum were lower than the CS1 values for both methods, and the value based on position of the mutations indicated significance at the *P* = .10 level for the CS3 sample. Furthermore, inspection of the spectra (Figure 3) revealed that expansion and contraction of the GGGGG pentanucleotide at positions 199–203 was strongly decreased in strain CS3 relative to CS1 and wild type (1 event versus 19 and 28, resp.). Other mutations at this site were not affected in strain CS3, however, and the other mononucleotide runs in *pyrE* seemed to expand and contract with comparable frequency in the three strains (Figure 3). In addition, strain CS3 exhibited a hotspot for G:C to A:T transition mutations at nt353 in the *pyrE*_Sac_ coding sequence (Figure 3(b)).

A final set of comparisons focused on the increased yield of recombinants from *S. acidocaldarius* matings that results when either or both parental strains are irradiated with UV-C before conjugation. The magnitude of this stimulation of marker exchange (SME) increases with the dose of UV and other DNA-damaging agents and decreases with the length of time that cells are held at physiological temperature before being allowed to pair [8]. SME and its decay thus provide quantifiable genetic consequences of DNA damage, consistent with an intermediate of DNA repair that stimulates DNA transfer, recombination, or both [8]. To test for effects on induction or decay of SME caused by disruption of Saci_1227 or Saci_1096, we chose a pair of *pyrE* mutations represented both in a disrupted and wild-type *S. acidocaldarius* and used those pairs to quantify marker exchange after UV exposure (Table 1).

**Figure 4:**
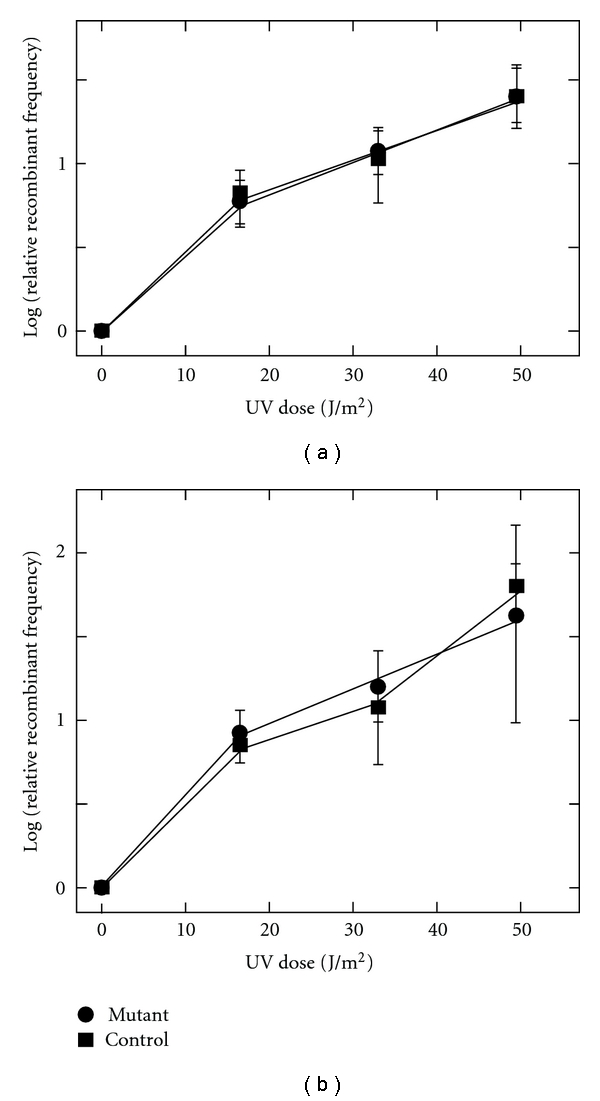
Stimulation of marker exchange (SME) by UV. Relative frequency of recombinants in response to indicated UV fluence UV was from an unfiltered germicidal lamp and the doses correspond to the short-wavelength portion of the output (*λ* < 300 nm). (a) CS1 (circles) versus DG185 (squares). (b) CS3 (circles) versus DG185 (squares).

**Figure 5:**
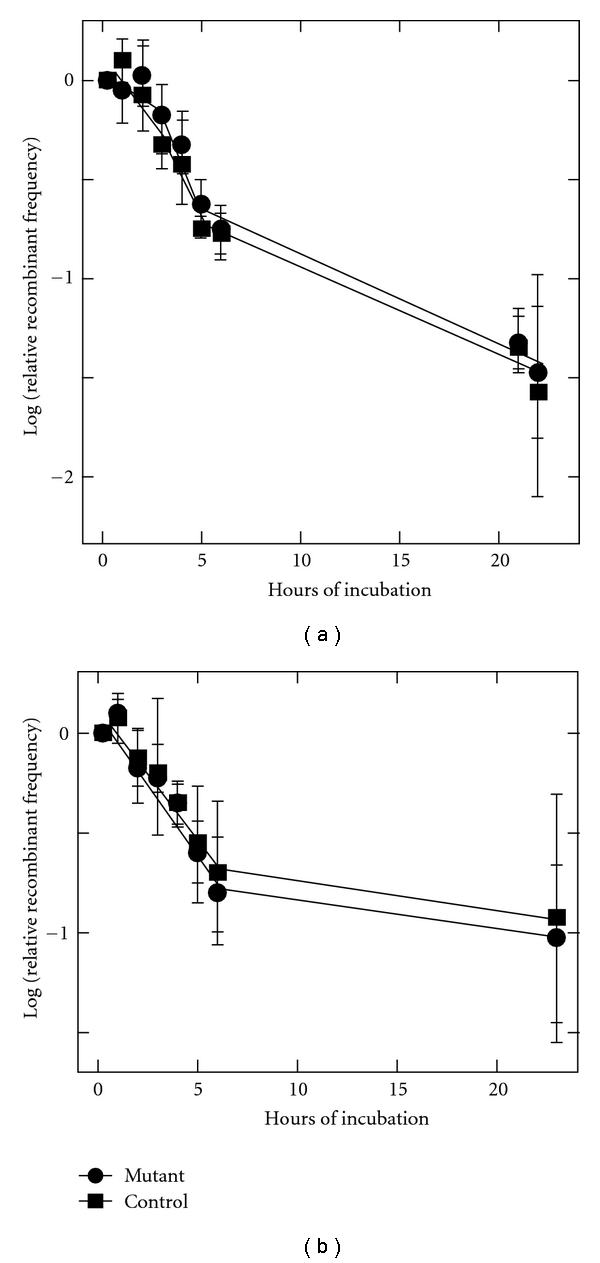
SME Decay. Frequency of recombinants is plotted as a function of time at 70°C after UV (33 J/m^2^), but before cells were mated (see Section 2). Symbols are as in Figure 4.

Using this approach, we first compared the magnitude of SME as a function of UV dose in each disruptant to that of wild-type *S. acidocaldarius* (Figure 4). The results indicate no obvious impact of either the Saci_1227 or Saci_1096 gene disruption. Recombinant yields from the UV-treated mutants were generally higher than the control, but the differences were smaller than the standard deviation at each UV dose. Similarly, the kinetics of SME decay suggested only slightly slower decay in the Phr^−^ strain, and the apparent difference was less than the standard deviation at each time point (Figure 5).

## 4. Discussion

The ORFs Saci_1096 and Saci_1227 represent a class of genes that have strategic importance for elucidating DNA repair and related transactions in hyperthermophilic archaea. The encoded proteins are related to enzymes that mediate known DNA repair processes in other organisms, raising the possibility that they may participate in repair of DNA in hyperthermophilic archaea as well. Most of these genes are not essential for viability in model organisms, and mutants completely lacking gene function have been isolated and characterized, providing phenotypes for comparison to corresponding mutants in hyperthermophilic archaea. We were able to disrupt Saci_1227 and Saci_1096 with a selectable biosynthetic gene and evaluate the functional relevance of these gene products to DNA repair *in vivo* with various quantitative assays. The results support four conclusions, described below.

### 4.1. The UVDE Homologue Encoded by Saci_1096 Does Not Play a Critical Role in the Dark Repair of UV Damage in *S. acidocaldarius* DNA

Disrupting this gene did not change the quantitative impact of UV-C radiation on viability, the increased yield of recombinants from conjugation (SME), or the loss of this increase with time under physiological conditions (SME decay). The Saci_1096 disruptant also failed to exhibit obvious sensitivity toward greek letter gamma radiation or several diverse DNA-damaging chemicals. The lack of measurable sensitivity was not due to retention of an intact copy of the gene, and contrasts with the corresponding results from UVDE mutants of model organisms. Specifically, in both the fission yeast *S. pombe* and the radioresistant bacterium *D. radiodurans*, disrupting the gene homologous to Saci_1096 caused an obvious decrease in UV survival relative to the control [11, 12, 31].

Questions concerning the function of Saci_1096 are also raised by the infrequent occurrence of such UVDE homologues (members of protein family PF 03851 [32]) among archaea. Besides *S. acidocaldarius*, which is the only crenarchaeote so far known to encode one of these proteins, archaeal PF 03851 members occur only in *Halococcus marismortui*, *Haloquadratum walsbyi*, *Methanococcus maripaludis*, one uncultured halophile, and one uncultured methanogen [32]). It should be noted that we cannot exclude the possibility that Saci_1096 has functional redundancy with another DNA repair function in *S. acidocaldarius*, as mutants lacking other relevant repair pathways are not yet available. Such redundancy does not mask UV-sensitive phenotypes in *D. radiodurans* or *S. pombe*, however [11, 12, 31]. It must also be considered that the Saci_1096 protein may have a role in DNA metabolism that does not involve repair of UV photoproducts or other environmentally induced DNA damage but does affect some aspect of DNA replication. We observed evidence for this in the *pyrE* mutation spectra, as one frameshift hotspot was strongly attenuated in the Saci_1096 disruptant, and a new hotspot for transition mutations was created. The mechanistic basis of this effect remains unclear, however, as other mutations occurred at this site in CS3, whereas frameshift mutagenesis at other similar sites in *pyrE* seemed unaffected.

### 4.2. Saci_1227 Encodes a Functional DNA Photolyase

Disruption of Saci_1227 produced an *S. acidocaldarius* strain that exhibited negligible photoreactivation, making this gene product the first DNA repair protein of hyperthermophilic archaea to be identified by a combination of biochemical, functional, and genetic criteria. Known photolyases provide the highest BLASTP scores to the *S. acidocaldarius* protein and homologous proteins of related archaea, and purified *S. tokodaii* photolyase has been confirmed to reverse CPDs *in vitro* [9]. In addition, *S. acidocaldarius* exhibits biological photoreactivation [10], and intact Saci_1227 is required for this response, as shown in the present study. The *S. acidocaldarius* photolyase action spectrum [10] resembles those of the absorbance spectrum of the *S. tokodaii* photolyase (i.e., shoulders at about 400 and 420 nm, and an abrupt cutoff at 500 nm) [9], and has an even closer similarity to the action spectrum of the *Thermus thermophilus* DNA photolyase determined *in vitro* [33]. In both the *S. tokodaii* and *T. thermophilus* enzymes, these spectral characteristics correlate with two bound flavins: one that promotes reversible single-electron transfer to the archaeal pyrimidine dimer and the other that serves as an antennary chromophore. The similar functional, structural, and spectral properties of the Saci_1227 protein thus suggest that it too may use a second flavin as the antennary pigment.

### 4.3. The *S. acidocaldarius* DNA Photolyase Does Not Play a Significant Role in Dark Repair of UV Photoproducts

Although hyperthermophilic archaea have functional systems of homologous recombination and translesion DNA synthesis, which are expected to contribute to tolerance of UV photoproducts, gene inventories of these organisms do not suggest how UV photoproducts and other large, helix-distorting lesions can be excised [5]. The central uncertainty regarding excision repair in these archaea concerns initial recognition of the UV photoproducts by the repair system. *Sulfolobus* spp. encode homologues of several eukaryotic NER proteins, but none of the canonical damage-recognition proteins known from eukaryotes or bacteria. Furthermore, *S. solfataricus* transcription does not influence the kinetics of UV photoproduct repair, implying that RNA polymerase does not serve as a sensor for these lesions in *Sulfolobus* [6, 7]. The bulk of the available evidence thus suggests that dark repair of UV damage in *Sulfolobus,* and perhaps in other hyperthermophilic archaea, may be completed by homologues of eukaryotic NER enzymes, but it is initiated by yet-unidentified factors unrelated to the canonical bacterial (UvrABC) and eukaryotic (Rad4-Rad23-Rad14) damage-recognition proteins.

Since DNA photolyases bind specifically to UV photoproducts, the *Sulfolobus* enzymes could, in principle, serve as the damage recognition proteins for dark repair of UV-induced DNA damage in *Sulfolobus*; alternatively, this property may cause these light-dependent enzymes to interfere with such repair. Photolyases of bacteria and fungi have been seen to exert either positive or negative effects on NER of UV photoproducts, depending on the situation. *E. coli* photolyase, for example, stimulates the UvrABC system *in vitro* [13], whereas the yeast photolyase potentiates the lethal effect of various DNA-damaging agents by inhibiting NER *in vivo* [34].

Construction of Saci_1227 disruptants allowed us to test for an influence of photolyase on dark repair of UV photoproducts, regardless of whether this influence is positive or negative. Biological responses to UV-C that we observed to be equal in *phr* ^−^ versus *phr* ^+^ strains included killing and SME as a function of UV dose, sensitivity to DNA-damaging chemicals, frequency of spontaneous *pyrE* mutations, and the position and molecular nature of these mutations. The kinetics of SME decay appeared to be slightly slower in the Phr^−^ strain than in the control strains; such a result would be explained by accelerated incision at the DNA lesions, retarded resolution of a recombinogenic repair intermediate, or some combination of these two effects. However, the small magnitude of the observed response argues that the *Sulfolobus* photolyase neither assists nor inhibits the (yet unidentified) dark-repair mechanism(s) of *Sulfolobus* to a significant extent.

### 4.4. PCR Primers Can Target Gene Disruption in *S. acidocaldarius*

 Various genetic assays indicate that the homologous recombination system of *S. acidocaldarius* can operate on very short DNA sequences. For example, synthetic oligonucleotides can replace chromosomal mutations, including small deletions [18, 23], and recombination events readily segregate genetic markers spaced only a few bp apart [17, 19]. The present study demonstrated that short (40 to 50 bp) flanking sequences, incorporated into PCR product as 5′ extensions of primers, direct the site-specific integration of a selectable gene into the *S. acidocaldarius* genome. This method of gene disruption has played a strategic role in the high-throughput, systematic genetic analysis of other micro-organisms, notably* Saccharomyces cerevisiae* [20], and has similar potential for HA, as well.

The PCR-tailing technique of gene disruption also has certain disadvantages that should be acknowledged, including the fact that it generally consumes a selectable marker for each gene disrupted. This becomes significant for genetic analysis in *Sulfolobus* species, because few selectable markers are currently in use for these archaea. We were able to perform quantitative genetic tests on disruption mutants by restoring the native *pyrE*_Sac_ gene. Such “marker recycling” is made practical by the two counterdirectional selections that can be applied to *pyrE* and *pyrF* genes of *Sulfolobus *spp., but our procedure was laborious and other selectable markers generally do not have this versatility. Thus, alternative gene-disruption strategies, such as the integration/pop-out method used in *S. cerevisiae* and other microorganisms, including methanogens [35], merit continued development for *Sulfolobus* spp. and other hyperthermophilic archaea.

## Figures and Tables

**Table 1:** Strains, plasmids, and primers.

Designation	Genotype	Source or reference
*S. acidocaldarius* strains		

DG185	Wild-type	Obtained as ATCC 33909
MR31	*py* *rE*131_Sac_ (18-bp internal deletion)	[21]
LR10	Saci_1227::*pyrE*_Sso_^+^	Pyr^+^ transformant of MR31 (see text)
LR12	Saci_1096::*pyrE*_Sso_^+^	Pyr^+^ transformant of MR31 (see text)
DG251	Saci_1427::*pyrE*_Sso_^+^	Pyr^+^ transformant of MR31 (see text)
DG260	Saci_1227::*pyrE*_Sso_^−^,	Spontaneous FOA-resistant LR10
	*py* *rE*131_Sac_	
DG262	Saci_1096::*pyrE*_Sso_^−^,	Spontaneous FOA-resistant LR12
	*py* *rE*131_Sac_	
CS1	Saci_1227::*pyrE*_Sso_^−^	DG260 transformed to *pyrE*_Sac_^+^
CS3	Saci_1096::*pyrE*_Sso_^−^	DG262 transformed to *pyrE*_Sac_^+^
CS1mE	Saci_1227::*pyrE*_Sso_^−^	Spontaneous FOA-resistant CS1
	*py* *rE*_Sac_ ΔG199	
CS1m15	Saci_1227::*pyrE*_Sso_^−^	Spontaneous FOA-resistant CS1
	*py* *rE*_Sac_ A335T	
JDS8	*py* *rE*_Sac_ ΔG199	Spontaneous FOA-resistant DG185 (lab collection)
JDS28	*py* *rE*_Sac_ A335T	Spontaneous FOA-resistant DG185 (lab collection)
CS3m65	Saci_1096::*pyrE*_Sso_^−^	Spontaneous FOA-resistant CS3
	*pyrE*_Sac_ ΔC78	
CS3m78	Saci_1096::*pyrE*_Sso_^−^	Spontaneous FOA-resistant CS3
	*py* *rE*_Sac_ G203A	
DGm18	*py* *rE*_Sac_ ΔC78	Spontaneous FOA-resistant DG185 (lab collection)
DGm23	*py* *rE*_Sac_ G203A	Spontaneous FOA-resistant DG185 (lab collection)

*E. coli* plasmids		

pMJ03	SSV1-derived* Sulfolobus* shuttle vector	[24]
pPCBE12	3′ portion of Saci_1427 in MCS of pUC19	Phil Clark (unpublished)
pLK3a	*py* *rE*_Sso_ in MCS (XbaI site) of pNEB193	This work
pLK4a	*py* *rE*_Sso_ in *trpC* insert of pPCBE12	This work

PCR primers		

SsoPEAvrKpnr1	GCA GCC TAG GTA CCG ATA TGA GAG AGG TTT ATC CAT TGC	
SsoPEFKpnAvrf1	GCA CCT AGG ACG TGT CTT AAT CTC ACA AAG CCC TTA T	
SacPhr::cassf1		
CAACGAGACGAGAAAATGAAGGAAAATGCCTTGAATAAAGGAATTAAATTTACCGCACAGAGCGTAAGGAGAA		
SacPhr::cassr1		
ATCCACTAATTTTGTCGCAAAATACCTCTCTCCCAGTCTCCAGTCGACAATTATAGTCCTGTCGGGTTTCGCCA		
Saci1096 DTf3		
CACTTAGAATTATTCACTGTTGAGAGTAGGTTACGTATCCAAGTCTAGGGGTACCACGTGTCTTAATCTC		
Saci1096 DTr2		
GATTCACCTTAATAAATCCTCTAATCCAGTTTGTTTAACTCCTAATGCAGCTGGCACGACAGGTTTC		
Saci1227f	AAGAGAGACCACCGGTCTGAATGT	
Saci1227r	CCT TGGTCATGCCTTCGTGCTAAA	
Saci_1227internalF	CACCATTTTACACTAAAGCAAGAG	
Saci_1227internalR	CGACCAACATTCTCACTCTTC	
NXSaci1096f	GAACGCCGGCTCGAGCAAGAGGGTCAAGTCGATAATTGG	
NXSaci1096r	GAGGCCGGCTCGAGGGGCGTTTGGTATACTGTTCTATC	
Saci_1096internalF	GAGTGCTTAAAGTCTCTTCCTC	
Saci_1096internalR	CTTATCCTTCACCTCAAGCATG	

**Table 2:** Sensitivity of Saci_1096 mutant to DNA-damaging agents^a^.

Damaging chemical	Type of damage	DG185 (con)	DG251 (con)	LR12
cisplatin* (cis* platinum(II) diammine dichloride)	Intra- and interstrand crosslinks	37.5 ± 0.0	37.5 ± 0.0	37.5 ± 0.0
MNNG (N-methyl N′-nitro N-nitrosoguanidine)	Base methylation	43.8 ± 28.6	43.8 ± 28.6	43.8 ± 28.6
Mechlorethamine (2-chloro-N-(2-chloroethyl)-N-methyl-ethanamine)	Intra- and interstrand crosslinks	250.0 ± 86.6	250.0 ± 86.6	250.0 ± 86.6
Metronidazole (2-(2-methyl-5-nitro-1H-imidazol-1-yl)ethanol)	Bulky adduct	2.5 × 10^3^ ± 0	(no data)	2.5 × 10^3^ ± 0
4-nitroqunoline N-oxide	Bulky adduct	0.4 ± 0.17	0.5 ± 0.17	0.5 ± 0.17
Butadiene diepoxide	Intrastrand crosslinks	100 ± 43.3	100 ± 43.3	100 ± 43.3
Hydrogen peroxide	Oxidation and strand breaks	93.8 ± 44.2	93.8 ± 44.2	93.8 ± 44.2

^**a**^ The average MIC values ± standard deviation (*μ*g/mL) were calculated from 3 replicates, except for hydrogen peroxide (average of two replicates).

**Table 3:** Molecular nature of spontaneous mutations in disruptants versus wild type.

	CS1	CS3	DG185
Frequency of mutations^1^			
BPS	0.25	0.36	0.26
Transitions	0.13	0.21	0.17
Transversions	0.11	0.15	0.09

Frameshift	0.69	0.55	0.62
plus 1	0.44	0.41	0.40
minus 1	0.21	0.14	0.22
plus 2	0.02	0.00	0.00
minus 2	0.02	0.00	0.00

Del/Ins (≥3 bp)	0.07	0.09	0.12
Deletions	0.02	0.03	0.02
Insertions	0.05	0.06	0.10

*P* values for hypergeometric statistical analyses^2^			

Criteria			
Position of mutations	.94	.10	—
Type of mutations	.88	.45	—

**^1^**Number of mutations identified in each strain: CS1, 61; CS3, 78; DG185, 107.

**^2^**Estimated *P* values from hyper-geometric tests comparing the mutation spectra of CS1 and CS3 to that of wild type using two criteria, that is, position of mutation regardless of type, and types of mutation regardless of position. For the former, each indel was considered to be located at the first nucleotide affected. The latter used the following 13 categories of mutations: eight frameshifts (+/−A, C, G, T), four base-pair substitutions (A:T → T:A; C:G → G:C; A:T ←→ G:C; A:T ←→ C:G), and indels ≥3 bp.
